# Supplementary material for: Protocol for the ‘Supporting Young Cancer Survivors who Smoke’ study (PRISM): Informing the development of a smoking cessation intervention for childhood, adolescent and young adult cancer survivors in England
Source: PLoS One. 2024 May 15;19(5):e0299321. doi: 10.1371/journal.pone.0299321 (PMC11095735; doi:10.1371/journal.pone.0299321)
Supplement: S1 File — (PDF) [file pone.0299321.s001.pdf]

Draft topic guide

SUPPORTING YOUNG CANCER SURVIVORS WHO SMOKE

FOR INFORMATION: *This is a topic guide only. The participant will be free to dictate the direction and content of the interview in relation to their views and experiences. The early interviews and early stages of analyses may identify additional issues which may be useful to follow-up in subsequent interviews. Therefore, the topic guide will be used flexibly and will evolve as the study progresses.*

Question	Rationale
**Are you able to just say a little about yourself, what you do, who you live with, your hobbies/things you enjoy doing?**	Introduction
**It would be useful to know a little about your illness history if that is ok?**<br>- How old you were when you were diagnosed?<br>- Can you remember what treatment you received?<br>- Do you know how long it has been since you finished treatment?	Background information
**Do you still see any healthcare professionals for follow-up care for your health after cancer?**<br>- If so, who with (GP/consultant)?<br>- What do you think the purpose of this follow-up is?<br>- Do you have regular appointments at the hospital for scans, or other procedures?	Background information
**Exploring smoking/vaping status and knowledge**	
**Could I please confirm your current smoking/vaping status?**<br>- Are you smoking/vaping at the moment/recently given up?<br>- If smoker/vaper, how often/how much would you say you smoked/vape?<br>- What are your views on your smoking/vaping?<br>- If recently given up, when?	Smoking/vaping status
**How old were you when you started smoking/vaping?** (before, during, after treatment)<br>- Can you remember how you started?<br>- Can you remember your reasons for starting?	Smoking/vaping history; reasons for starting
**If someone like yourself wanted to quit smoking/vaping, do you know where they could get help and advice to do this?**<br>- How do you know about these services/resources?<br>- Have you ever used any of them? Been referred?<br>- What was your experience of using them?	Knowledge of available services to help quitting
**What do you know about the strategies that people can use to help them quit smoking/vaping?**	Knowledge of strategies to quit

<table>
<tr><td colspan="2">IF PARTICIPANT IS A CURRENT SMOKER AND/OR CURRENT VAPER - MOVE TO SECTION A
IF PARTICIPANT IS AN EX-SMOKER - MOVE TO SECTION B</td></tr>
<tr><td>SECTION A – CURRENT SMOKER OR CURRENT VAPER</td><td></td></tr>
<tr><td>So, have you ever tried to quit?
- If yes, how did you try to quit/what strategies did you use?
- How long did you manage to quit for?
- What were the main reasons for you wanting to stop?
- How many times do you think you have tried to quit?
- What do you feel were the main reasons that you started smoking again?</td><td>Attempts to quit

Behavioural regulation</td></tr>
<tr><td>Is quitting something you are considering at the moment?

If yes, how motivated would you say you are to quit?
- What would you say is your main motivation/the main reason to quit?
- Is there a timescale you have – a time when you would like to quit by?
- Are there any other things that you like or need to do that get in the way of quitting?

If no, why?
- Is it something you may consider in future?
- What about cutting down?
- What would you say is the main reason for not wanting to quit?</td><td>Motivation to quit/intention to quit - goals</td></tr>
<tr><td>If you decided to quit for good, how easy do you think it would be for you to do it?
- What things could make it more difficult, if anything?
- What would help you overcome these difficulties?
- What would make it easier/help you, if anything? Why?
- How much control do you think you have over your smoking?
- Would you say you are addicted to cigarettes? What makes you say this?</td><td>Beliefs about capabilities to quit</td></tr>
<tr><td>How optimistic are you that you will be able quit in the future?</td><td>Optimism</td></tr>
<tr><td>Are there any skills you would like to learn that would help you to quit/cut down/remain abstinent?</td><td>Skills</td></tr>
<tr><td>So you’ve smoked/vaped for X years, how much would you say that being a smoker/vaper is part of your personality or who you are?</td><td>Social professional role/identity</td></tr>
<tr><td>How much time during the day do you find yourself thinking about smoking/vaping?
Is there anything that distracts you and makes it easier not to think about smoking/vaping?</td><td>Memory, attention and decision processes</td></tr>
<tr><td>Does your mood influence whether you have a cigarette/use your vape or not? (angry, depressed, frustrated, stressed, bored, nervous)

And when you do smoke/vape, do you find it influences your mood/how you feel all?</td><td>Emotions</td></tr>
</table>

**Would you say that there are any advantages/benefits to smoking/vaping?** (health, personal, social, financial)<br>- Is there anything that you get from smoking that you would miss if you stopped?<br>- Are there any disadvantages to smoking/vaping?<br>- Would you say the advantages outweigh the disadvantages?<br><br>**Would you say that there are any advantages to quitting smoking/vaping?** (health, personal, social, financial)<br>- Are there any disadvantages of quitting?<br>- Would you say the advantages outweigh the disadvantages?	Beliefs about consequences of smoking and quitting
**Where do you usually smoke/vape?** Why here?<br>- Is there anything in your surroundings/where you spend your time that have helped you quit/cut down?<br>- On the other hand, is there anything about your surroundings/where you spend time that have stopped you from quitting/ cutting down?<br><br>**How does the cost of smoking/vaping influence your desire to quit/cut down?**	Environmental context and resources
**Do the people you spend time with/socialise with smoke/vape or not?** (friends, family, partner, work mates)<br>- How does this influence your smoking/attempts to quit/attempts to cut down? (temptation, offered cigarettes)<br><br>**Do your family/partner/friends have views about your smoking/vaping?**<br>**What would they think about you quitting/trying to quit smoking/vaping?**<br>- Would you say their views influence you?<br>- Is there anyone who encourages you to smoke? Anyone or supports you to quit smoking? How do they encourage/support you?<br><br>**Generally how many people (same sex and age; cancer history) do you think smoke?**	Social influences<br><br>Family/friends
**Has your oncologist or nurse specialist ever talked to you about your smoking/advised you to quit?**<br>- If yes, what did they say? When was this? Once off or regular? Did they offer you any support to quit?<br>- Does them talking to you about smoking/giving advice make you think about your smoking/quitting?<br>- Should smoking be talked about at clinic?<br><br>- If no, do they know that you smoke/vape?	Social influences<br>Intervention of cancer care team
**Has your GP ever talked to you about your smoking/advised you to quit?**<br>- If yes, what did they say? When was this? Once off or regular? Did they offer you any support to quit?<br>- If no, do they know that you smoke?	Social influences<br>Intervention of GP

Brown et al., (2024). Protocol for the ‘Supporting Young Cancer Survivors who Smoke’ study (PRISM): Informing the development of a smoking cessation intervention for childhood, adolescent and young adult cancer survivors in England.

**Do you think that people who want to quit smoking/vaping, should be offered help and support to quit?**<br>- Who should provide help? When should they provide help?<br>- What kind of help would be most useful? What would work/wouldn't work?<br>- Views on E-cigarettes, medication?	
**Closing words**<br>- *Ask if there was anything else the participant wishes to discuss or feels is important.*<br>- *Ask if there is anything they want to ask about the study.*<br>- *Give interview debrief sheet and thank them for their participation.*	
**SECTION B – EX-SMOKER**	
**So, when did you quit?**<br>- How did you try to quit/strategies used? What helped you to stop smoking?<br>- Was this the first time you had tried to quit?	Attempts to quit & strategies used
**These questions are to gain your views on what may influence your smoking behaviour, or your views of quitting.**	
**How motivated would you say you were to quit?**<br>- What would you say is your main motivation/the main reasons to quit?<br>- Are there any other things that you like or need to do that get in the way of quitting/cut down/ remain abstinent?	Motivation to quit/intention to quit - goals
**When you had decided that you wanted to quit, how easy did you think it would be to do?**<br>- How easy did you find it?<br>- What were the main difficulties you came across? (social, physical<br>- How did you overcome these difficulties?<br>- Was there anything that helped you? What were they?<br>- Would you say you that you were addicted to cigarettes?<br>- Was there a point it got easier? Why was this?	Beliefs about capabilities to quit/remain abstinent
**How much control do you think you have over your smoking?**<br>**How optimistic are you that you will be able to remain abstinent?**	Optimism
**From your experience of quitting, do you think there any skills you would like to learn that would help people to quit?**<br>**What about skills for people to remain abstinent?**	Skills
**So you smoked for X years, how much would you say that being a smoker was part of your personality or who you were?**<br>- Has this changed now you have quit?<br>- Has quitting changed how you view yourself at all?	Social professional role/identity

**How much time during the day do you find yourself thinking about smoking/vaping?**<br>**Is there anything that distracts you and makes it easier not to think about smoking/vaping?**	Memory, attention and decision processes
**Did your moods influence your smoking behaviour at all?**<br>- How has this changed since quitting?	Emotions
**Would you say that there are any advantages to smoking?** (health, personal, social, financial)<br>- Is there anything that you get from smoking that you would miss if you stopped?<br>- Are there any disadvantages to smoking?<br>- Would you say the advantages outweigh the disadvantages?<br><br>**Would you say that there are any advantages to quitting smoking?** (health, personal, social, financial)<br>- Are there any disadvantages of quitting smoking?<br>- Would you say the advantages outweigh the disadvantages?	Beliefs about consequences of smoking and quitting
**Where did you usually smoke?** Why here?<br><br>- Was there anything in your surroundings/where you spent your time that helped you quit?<br>- On the other hand, was there anything about your surroundings/where you spent time that stopped you from quitting?<br><br>**Did the cost of smoking influence your desire to quit?**	Environmental context and resources
**Do the people you spend time with/socialise with smoke or not?** (friends, family, partner, work mates)<br>- How did this influence your smoking/attempts to quit? (temptation, offered cigarettes)<br><br>**Did your family/partner/friends have views about your smoking?**<br>**What did they think about you quitting/trying to quit smoking?**<br>- Would you say their views influence you?<br>- Is there anyone who encourages you to smoke? Anyone or supports you to quit smoking? How do they encourage/support you?<br><br>**Generally how many people (same sex and age; cancer history) do you think smoke?**	Social influences<br><br>Family/friends
**Has your oncologist or nurse specialist ever talked to you about smoking/did they advise you to quit?**<br>- If yes, what did they say? When was this? Once off or regular? Did they offer you any support to quit?<br>- Did<br>- Did the advice/support provided by your oncologist make it easier or more difficult to quit/cut down?	Intervention of cancer care team

- If no, do they know that you smoked?	
**Has your GP ever talked to you about smoking/did they advise you to quit?**<br>- If yes, what did they say? When was this? Once off or regular? Did they offer you any support to quit?<br>- If no, do they know that you smoked?	Intervention of GP
**Do you think that people who want to quit smoking, should be offered help and support to quit?**<br>- Who should provide help? When should the provide help?<br>- What kind of help would be most useful?	
**Closing words**<br>- *Ask if there was anything else the participant wishes to discuss or feels is important.*<br>- *Ask if there is anything they want to ask about the study.*<br>- *Give interview debrief sheet and thank them for their participation.*	
